# Supplementary Addendum to “Radiation dose of digital radiography (DR) versus micro-dose x-ray (EOS) on patients with adolescent idiopathic scoliosis: 2016 SOSORT- IRSSD “John Sevastic Award” Winner in Imaging Research”

**DOI:** 10.1186/s13013-017-0148-5

**Published:** 2018-01-08

**Authors:** Steve C. N. Hui, Winnie C. W. Chu

**Affiliations:** Department of Imaging and Interventional Radiology, Prince of Wales Hospital, The Chinese University of Hong Kong, Sha Tin, Hong Kong

**Keywords:** Adolescent idiopathic scoliosis, X-ray simulation, Biplanar X-ray

## Abstract

Regarding the publication entitle “Radiation dose of digital radiography (DR) versus micro-dose x-ray (EOS) on patients with adolescent idiopathic scoliosis: 2016 SOSORT- IRSSD “John Sevastic Award” Winner in Imaging Research” in *Scoliosis and Spinal Disorders*, we would like to provide more details to readers about the dose calculated by simulation using PCXMC 2.0. In this study, the data and results are correct based on the given parameters and calculation provided in the manuscript. In the simulation of EOS micro-dose, only a 0.1 mm copper filter was applied. We agree with the suggestion from Dr. Pedersen and colleagues that the inclusion of a 1.5 mm aluminum filter together with the 0.1 mm copper reflects more realistic representation of X-ray filtration which would improve the accuracy of the simulation. We believe that this supplementary addendum would be beneficial to other researchers who are planning to conduct a similar study.

We are writing to provide additional details regarding the article published by Hui et al. (Radiation dose of digital radiography (DR) versus micro-dose x-ray (EOS) on patients with adolescent idiopathic scoliosis: 2016 SOSORT- IRSSD “John Sevastic Award” Winner in Imaging Research, Scoliosis Spinal Disord. 2016 Dec 29;11:46. doi: 10.1186/s13013-016-0106-7.). X-ray filters using copper or aluminum eliminate undesirable low-energy photons which affect the effective dose due to the change of energy spectrum of the X-ray beam and tissue absorption. The dose calculated by simulation using PCXMC 2.0 in this article was solely based on using parameters provided in Table 2 of Hui et al. 2016 which included only a 0.1 mm copper filter; the inherent 1.5 mm aluminum filter was omitted. We recalculated effective dose with the additional (aluminum) filtration, and this showed that the average effective dose for the EOS micro-dose protocol would be increased from 2.6 μSv as shown in Table 3 of Hui et al. 2016 to 3.1 μSv similar to the results obtained by Dr. Pedersen and colleagues as both copper and aluminum filters were applied (P. Pedersen, A. Greval and S. Eiskjær, personal communication, July 12, 2017). We suggest using both filters in simulations to determine results that best reflect real circumstance if other investigators plan to conduct similar studies.

